# Identification of Duplication Downstream of *BMP2* in a Chinese Family with Brachydactyly Type A2 (BDA2)

**DOI:** 10.1371/journal.pone.0094201

**Published:** 2014-04-07

**Authors:** Xudong Liu, Linghan Gao, Aman Zhao, Rui Zhang, Baohu Ji, Lei Wang, Yonglan Zheng, Bingfang Zeng, Robert K. Valenzuela, Lin He, Jie Ma

**Affiliations:** 1 Department of Orthopaedic Surgery, Shanghai Sixth People's Hospital Affiliated to Shanghai Jiaotong University, Shanghai, China; 2 Bio-X Institutes, Key Laboratory for the Genetics of Developmental and Neuropsychiatric Disorders (Ministry of Education), Shanghai Jiao Tong University, Shanghai, China; 3 Department of Genetics and Molecular Biology, Xi'an Jiaotong University School of Medicine, Xi'an, Shaanxi, China; 4 Department of Psychiatry, School of Medicine, University of California San Diego, San Diego, California, United States of America; 5 Department of Medicine, the University of Chicago, Chicago, Illinois, United States of America; 6 Human Genetics, Genome Institute of Singapore, Agency for Science, Technology and Research (A*STAR), Singapore, Singapore; 7 Xi'an Hong Hui Hospital, the Affiliated Hospital of Xi'an Jiaotong University School of Medicine, Xi'an, Shaanxi, China; Central China Normal University, China

## Abstract

Brachydactyly type A2 (BDA2, MIM 112600) is characterized by the deviation and shortening of the middle phalange of the index finger and the second toe. Using genome-wide linkage analysis in a Chinese BDA2 family, we mapped the maximum candidate interval of BDA2 to a ∼1.5 Mb region between D20S194 and D20S115 within chromosome 20p12.3 and found that the pairwise logarithm of the odds score was highest for marker D20S156 (Zmax = 6.09 at θ = 0). Based on functional and positional perspectives, the bone morphogenetic protein 2 (*BMP2*) gene was identified as the causal gene for BDA2 in this region, even though no point mutation was detected in *BMP2*. Through further investigation, we identified a 4,671 bp (Chr20: 6,809,218–6,813,888) genomic duplication downstream of *BMP2*. This duplication was located within the linked region, co-segregated with the BDA2 phenotype in this family, and was not found in the unaffected family members and the unrelated control individuals. Compared with the previously reported duplications, the duplication in this family has a different breakpoint flanked by the microhomologous sequence *GATCA* and a slightly different length. Some other microhomologous nucleotides were also found in the duplicated region. In summary, our findings support the conclusions that *BMP2* is the causing gene for BDA2, that the genomic location corresponding to the duplication region is prone to structural changes associated with malformation of the digits, and that this tendency is probably caused by the abundance of microhomologous sequences in the region.

## Introduction

The term brachydactyly (BD) is derived from the ancient Greek (brachy-: short; dactylos: digit). This term indicates the shortening of digits due to abnormal development of the phalanges and/or the metacarpals and may also be accompanied by other hand malformations, such as syndactyly, polydactyly, reduction defects, or symphalangism [1]. One of the most commonly used classifications of brachydactyly based on anatomical grounds was provided by Bell [2] and further elaborated by Temtamy and McKusick [3]. BD can occur either as an isolated malformation or as part of a complex malformation syndrome. The isolated BDs are classified clinically into BDA-E and three other subgroups [3]. BD types have certain overlapping features, including hypoplasia/aplasia of phalanges and abnormal interdigital joint formation, suggesting that the formations of phalanges and joints are linked on a developmental and molecular basis.

In recent years, most of the isolated brachydactylies have been characterized at the molecular level, and these studies have provided new insights into the mechanisms of joint and digit development [4]. BDA1 (MIM 112500) is characterized by the shortening or absence of the middle phalanges and is associated with mutations in the Indian hedgehog gene (*IHH*) or chromosome 5p13.3-p13.2 [5], [6], [7], [8], [9]. BDB1-2 (MIM 113000; MIM 611377) is characterized by terminal deficiency of the fingers and toes and mutations that have been identified in an orphan receptor tyrosine kinase ROR2 or in NOGGIN (*NOG*) [10], [11], [12], [13]. BDC (MIM 113100) can present with a range of anomalies (from shortening to hyperphalangy of the middle phalanges of the index, middle, and little fingers) and is caused by mutations affecting growth/differentiation factor 5 (*GDF5*) [14]. Two mutations of *HOXD13* are associated with distinctive limb phenotypes in which brachydactylies of specific metacarpals, metatarsals, and phalangeal bones are the most constant features and exhibit overlap with brachydactyly types D (MIM 113200) and E (MIM 113300) [15].

BDA2 (MIM 112600) was described first by Mohr and Wiredt in a large Norwegian kindred of Danish descent [16]. Patients afflicted with BDA2 display hypoplasia/aplasia of the middle phalanx of the second and, sometimes, fifth fingers. Common to all BDA2 families are the deviation and shortening of the middle phalange of the index finger and/or the second toe. BDA2 is genetically heterogeneous. Lehmann *et al.* found that BDA2 is caused by missense mutations in bone morphogenetic receptor 1 b (*BMPR1B*), a type I transmembrane serine-threonine kinase, and that three different missense mutations exist in three German families [17], [18]. The second BDA2-causing gene, *GDF5*, was identified in the descendants of the original BDA2 family by Seemann *et al*. and Kjaer *et al*. [19], [20], [21]. Recently, we discovered a Chinese BDA2 family, but no mutation was found either in the *BMPR1B* or *GDF5* loci in the genetic analysis, suggesting additional genetic heterogeneity of this trait. Therefore, we performed a genome-wide scan using 403 microsatellite markers in our BDA2 family.

More recently, duplications downstream of *BMP2* (NM 001200) were identified in two studies; one study comprised samples from two European families (∼5.9 kb and ∼5.5 kb), and the other study comprised samples from a Chinese family (∼4.6 kb) [22], [23]. In our current study, we found a 4,671 bp duplication downstream of *BMP2* (Chr20: 6,809,218–6,813,888) associated with BDA2 in our Chinese family that partially overlaps with the previously reported duplications but has a different breakpoint flanked by the microhomologous sequence *GATCA*. Our finding supports the hypothesis that the genomic location corresponding to the duplication region is prone to structural changes associated with malformation of the digits and that this tendency is probably caused by the abundance of microhomologous sequences in the region.

## Results

### Clinical phenotypes

**Figure 1:**
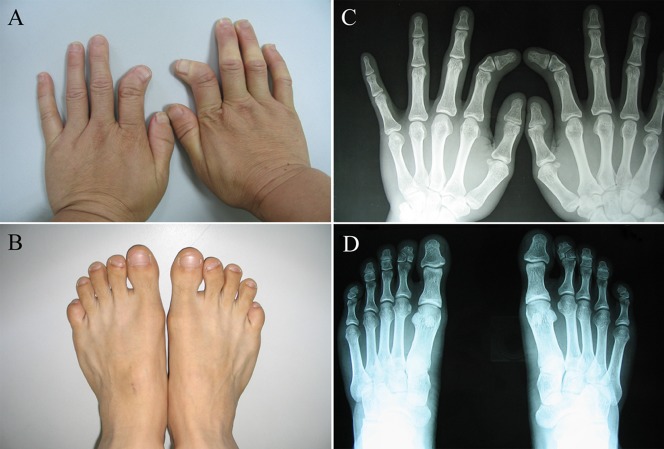
Phenotype of BDA2. A and B: Short and medially deviated index finger (above) and second toe (below). C and D: Radiograph of the triangular-shaped middle phalanx of the index finger (above) and the second toe (below).

In this six-generation family, 19 affected and 10 unaffected individuals were clinically examined, and a subset of the affected individuals was selected for radiography. All of the affected individuals had the typical phenotypes of BDA2 characteristic of medially deviated and shortened index fingers and second toes with abnormal interdigital joint formation. Most of the affected adult individuals exhibited short stature. Triangular-shaped middle phalanges were observed in all of the affected individuals, as demonstrated in the photograph and X-rays of two affected adult (Figure 1). No sex-correlated characteristics were observed in the family.

The common characteristic of BDA2 caused by variants in different genes is that almost all of the patients showed shortened index fingers. The phenotypic differences consisted of some abnormal symptoms in other phalanges or metacarpals in some patients and a few severely affected individuals, indicating overlap with another type of brachydactyly, symphalangism, or syndactyly. In addition, few mutation carriers appeared to be clinically normal (Table S1). No variant-specific phenotypes were observed in the affected individuals.

### Genetic analyses of *BMPR1B* and *GDF5*

The sequence analyses of the known BDA2 causing genes, *BMPR1B* and *GDF5*, in four affected family members (III:2, IV:4, IV:12, and V:12 in the family) revealed no mutations. In the two-point linkage analysis, the LOD scores were calculated for microsatellite markers in the *BMPR1B* and *GDF5* loci, but none of the LOD scores exceeded 2.0 (at θ = 0) (data not shown).

### Genome-wide scan and linkage analyses

A two-point LOD score for 21 markers on chromosome 20p12.3 for the pedigree are summarized in Table 1. Seventeen adjacent markers provided a positive LOD score (θ = 0), and the locus for BDA2 was mapped between markers D20S867 and D20S851 within a region of ∼5.2 Mb, based on the UCSC Human Genome (Mar 2006). The pairwise logarithm of the odds scores was highest for marker D20S156 (Zmax = 6.09 at θ = 0).

**Table 1:** Two-Point LOD Score Obtained from the Linkage Analysis between the BDA2 Locus and Chromosome 20p12.3 in the Pedigree.

Marker	Physical	LOD Score AT θ =							*Z*_max_	θ
	Location^a^	0.0	0.01	0.05	0.1	0.2	0.3	0.4		
D20S842	2634204	−2.62	1.78	2.82	2.98	2.58	1.83	0.92	2.98	0.1
D20S867	3624718	−2.77	1.76	2.74	2.84	2.41	1.69	0.87	2.84	0.1
D20S889	3894953	3.48	3.41	3.12	2.75	1.99	1.21	0.48	3.48	0
D20S849	5142034	1.54	2.48	2.81	2.69	2.13	1.44	0.70	2.81	0.05
D20S882	5583098	5.79	5.68	5.25	4.70	3.53	2.30	1.03	5.79	0
D20S95	5664250	2.85	3.78	4.06	3.85	3.09	2.12	1.03	4.06	0.05
D20S905	5811610	3.69	3.64	3.39	3.06	2.33	1.51	0.66	3.69	0
D20S194	6090696	2.68	3.58	3.84	3.63	2.94	2.09	1.10	3.84	0.05
D20S192	6645008	2.61	2.56	2.37	2.11	1.56	1.01	0.48	2.61	0
D20S156	6648994	6.09	5.98	5.55	5.00	3.81	2.54	1.21	6.09	0
D20S892	6698074	5.36	5.27	4.88	4.38	3.32	2.20	1.05	5.36	0
D20S846	6712933	4.37	4.29	3.98	3.58	2.73	1.83	0.91	4.37	0
D20S59	6739353	5.88	5.77	5.34	4.78	3.59	2.31	0.99	5.88	0
D20S448	7110405	3.07	3.02	2.80	2.49	1.80	1.09	0.45	3.07	0
D20S602	7274075	3.98	3.88	3.52	3.09	2.25	1.40	0.58	3.98	0
D20S900	7309583	1.88	1.86	1.75	1.56	1.13	0.65	0.21	1.88	0
D20S115	7607867	2.40	2.39	2.28	2.07	1.52	0.87	0.28	2.40	0
D20S907	8005228	2.48	2.43	2.20	1.91	1.33	0.76	0.29	2.48	0
D20S879	8512858	2.18	2.20	2.18	2.05	1.63	1.12	0.56	2.20	0.01
D20S851	8809809	−3.31	0.31	0.98	1.15	1.03	0.72	0.36	1.15	0.1
D20S186	11471795	−7.09	−0.75	1.0	1.48	1.52	1.12	0.56	1.52	0.2

a UCSC Browser, Mar 2006; http://genome.ucsc.edu/cgi-bin/hgGateway.

**Figure 2:**
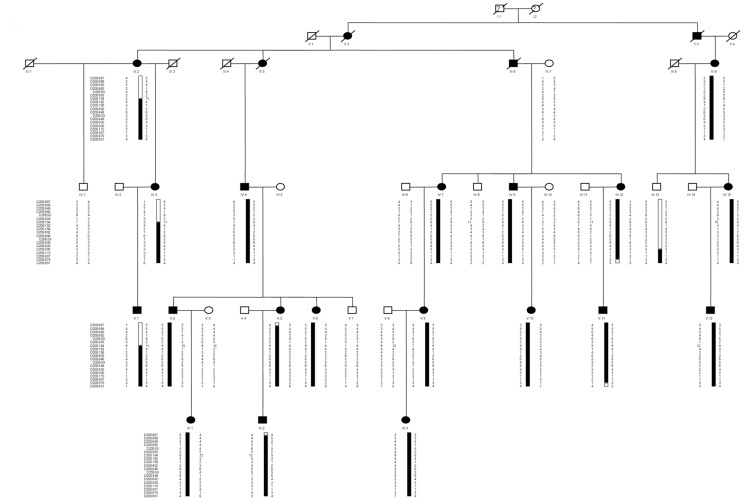
Pedigree structure and haplotypes of the family. The marker order was determined from the Marshfield map and the UCSC Human Genome database (Mar 2006). The open symbols indicate the unaffected individuals, the blackened symbols indicate the affected individuals, the squares indicate the males, and the circles indicate the females. The blackened bars indicate the chromosome region shared by the affected members of the pedigree.

The subsequent haplotypes were constructed using the CYRILLIC 2.1 software to define the interval of the linked region (Figure 2). The markers (19 total) used are listed in Table 1, and the haplotypes were checked through visual inspection. The recombination events between the BDA2 phenotype and the markers spanning the region of interest defined the smallest cosegregating region that included critical meiotic recombinants in the pedigree. Careful examination of the haplotypes confirmed that the disease-associated alleles co-segregated with the phenotype of BDA2 in the pedigree. A recombination event in individual II:2 placed the disease locus distal at D20S194 because the affected individual did not inherit the disease-linked D20S194 alleles observed in the family. The other recombination event in individual IV:13 placed the disease locus close to D20S115 because this unaffected male shared the disease-linked alleles of D20S115 with his affected mother. Hence, the maximal interval of linkage with the BDA2 phenotype is bordered by D20S194 (telomeric) and D20S115 (centromeric) within a region of ∼1.5 Mb. Inspection of this interval revealed only one gene, *BMP2*.

### Mutation and duplication analyses of *BMP2*

The sequence analysis of *BMP2* (i.e., all exons, introns, promoter region, 5′UTR, 3′UTR, and evolutionary conserved regions) showed no mutation.

**Figure 3:**
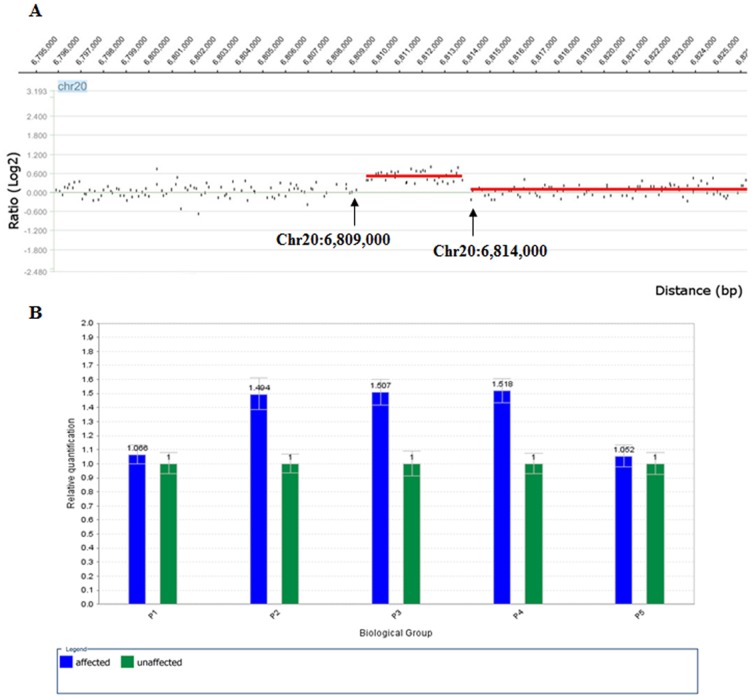
Microduplication on 20p12.3. A. Genomic profile of the microduplication as detected on the NimbleGen Human CGH 385K Chromosome 20 Tiling Array. The detected breakpoints are indicated by arrows. The duplication comprises ∼4.6 kb. The x axis shows the genomic positions on chromosome 20, and the y axis shows the log2 ratio. B. Microduplication confirmed by quantitative real-time PCR (Q-PCR) utilizing a ViiATM 7 Real-Time PCR system. The mean values of the relative quantification were exported from the ViiATM 7 software 1.0. The mean values and standard deviations (error bars) for each target amplicon relative to albumin, which was used as a two-copy reference gene, were calculated for eight affected (blue bars) and eight unaffected (green bars) individuals. The primers P1–P5 (P2–P4 are within the duplicate region, and P1 and P5 flank the region) were designed by Su *et al*. (2011). One duplicated allele plus one normal allele resulted in three copies of the amplicons of primers P2–P4 in the affected individuals and in a ratio of 1.5 relative to the two copies of the healthy control. The localization of the Q-PCR amplicons is illustrated in Figures 4A and 4B.

**Figure 4:**
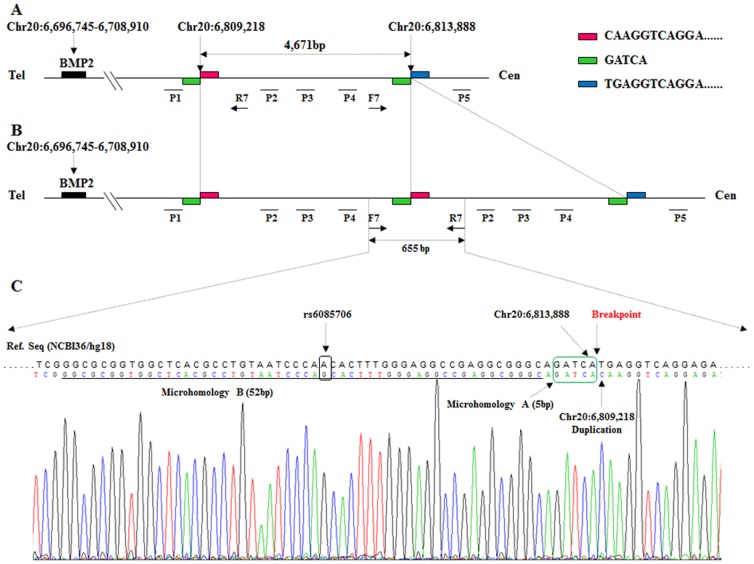
Results of long PCR, breakpoint identification, and positions of primers used. A. Structure of duplicated region in the unaffected members. F7 and R7: primers used in the breakpoint identification. P1–P5 are the primer positions used in the Q-PCR. The red and green bars indicate the breakpoint region. The numbers above the BMP2 denote the genomic position. Tel: telomere; Cen: centromere. B. Structure of duplicated region in patients. The duplicated region is between Chr20: 6,809,218 and 6,813,888. PCR using primers F7 and R7 amplified the 655 bp sequences spanning the breakpoint. C. Sequencing results of the breakpoint PCR product. The nucleotides of microhomology A (5 bp) are marked by a green box, and the nucleotides of upstream microhomology B (52 bp), including the rs6085706 SNP, are underlined. In addition, the microhomologous nucleotides *GTGACC* (7 bp) [22] and *TTGCAGTGAGC* (11 bp) [23] reported previously were also observed downstream of microhomology A (data not shown).

Using the Chromosome 20 array, a ∼5.0 kb duplication (Chr20: 6,809,000–6,814,000) was identified in an affected family member (IV:12) downstream of *BMP2* (∼110 kb; Figures 3A and 4A).

Utilizing Q-PCR with three primer pairs (P2–P4) within the duplicated region and two primers (P1 and P5) within the flanking sequences (Figures 3B and 4B), this duplication was confirmed in eight affected family members (IV:3, IV:4, IV:7, IV:12, IV:15, V:9, V:10, and VI:1) and was not found in eight unaffected family members (III:7, IV:1, IV:6, IV:8, IV:13, V:3, V:7, and V:8).

**Figure 5:**
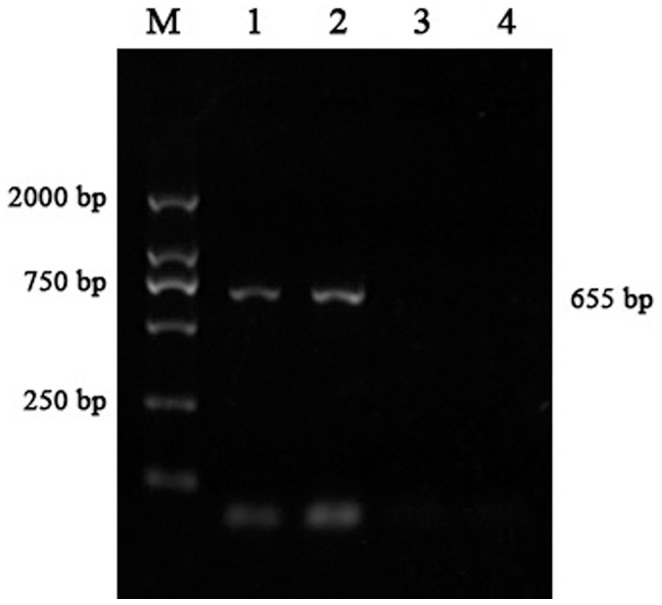
Agarose gel electrophoresis of PCR product with the breakpoint primers F7 and R7. There is a 655-bp band in the patient lanes (1 and 2) but no product in the lanes of the unaffected family member (3 and 4). M: marker.

**Figure 6:**
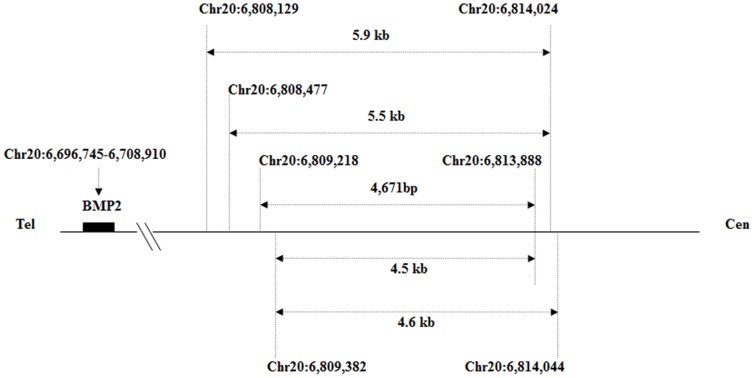
The chromosomal positions of the three previously reported duplications (Chr20: 6,808,129–6,814,024; Chr20: 6,808,477–6,814,024; and Chr20: 6,809,382–6,814,044) are indicated by the horizontal arrows. Comparison with the reported duplications shows that our duplication (Chr20: 6,809,218–6,813,888) overlaps with them but has different ends. Furthermore, the causal sequences of BDA2 could be narrowed down to 4,507 bp (Chr20: 6,809,382–6,813,888).

Using primers F7 and R7, a 655 bp PCR fragment was only produced in the patients (Figures 4B and 5). Moreover, through Q-PCR and breakpoint-PCR, this duplication was not found in 150 healthy control individuals excluding a common copy number variant (CNV). Through the direct sequencing of this 655 bp fragment in eight affected family members (IV:3: File S1; IV:4: File S2; IV:7: File S3; IV:12: File S4; IV:15: File S5; V:9: File S6; V:10: File S7; and VI:1: File S8), we identified a 4,671 bp duplication (Chr20: 6,809,218–6,813,888) downstream of *BMP2* and found that this duplication is flanked by the microhomologous sequences *GATCA* on the 5′ end (Chr20: 6,809,213–6,809,217) and 3′ end (Chr20: 6,813,884–6,813,888). Furthermore, in addition to the rs6085706 SNP, another microhomology (52 bp) could also be identified at the 5′ (Chr20: 6,809,160–6,809,211) and 3′ (Chr20: 6,813,831–6,813,882) flanking region of the 4,671 bp duplicated sequence. In addition, the microhomologous nucleotides *GTGACC* (7 bp) [22] and *TTGCAGTGAGC* (11 bp) [23] reported previously were also observed downstream of *GATCA*. The comparison with the previously reported duplications showed that the duplication identified in this study does have a different breakpoint, overlaps with the other three duplications, and differs in its 5′ and 3′ ends (Figure 6). Based on the above-described findings, we conclude that the fragment was duplicated and is associated with BDA2.

## Discussion

The family described in the current study has typical characteristics of BDA2 [1], [24], [25]. We excluded *BMPR1B* and *GDF5*, which were found to cause BDA2 in the previous studies [17], [18], [19], [20], [21], and mapped BDA2 to the locus within chromosome 20p12.3 because a strong linkage was observed between the BDA2 locus and the microsatellite markers in this chromosome region. The recombination events in individuals II:2 and IV:13 of the pedigree reduced the interval to a region of ∼1.5 Mb between D20S194 and D20S115.

Two factors enabled us to identify *BMP2* as the BDA2-causing gene in chromosome 20p12.3. First, the results of genome-wide scan showed that *BMP2* is the only known gene in the candidate interval, i.e., this gene is located at 6.697–6.709 Mb within the candidate interval (6.65–7.61 Mb on chromosome 20p12.3). It has been estimated that ∼25% of the human genome consists of long regions that contain no protein-coding sequences [26]. Some of these long regions contain regulatory sequences that act as large distances to control the expression of neighboring genes, and the sequence elements play critically important and conserved biological roles [27], [28], [29], [30]. Disruption of the long-range control of gene expression may cause disease [31]. *BMP2* was embedded in a long region with no known genes ∼0.6 Mb upstream and ∼1.1 Mb downstream that was linked with the phenotype of BDA2 in the pedigree. It has been reported that the transcription of *BMP2* is regulated by an enhancer region located 156.3 kb from the promoter of *BMP2* and that its noncoding sequence variants may influence *BMP2* transcription and thus may contribute to bone mass and osteoporosis [32], [33].

Second, *BMP2* has a functional role in the BMP signaling pathway similar to that of the previously identified genes, i.e., *BMPR1B* and *GDF5*, that cause BDA2 disease [17], [18], [19], [20], [21], [34]. The proteins of the transforming growth factor β (TGF-β) superfamily are involved in the process of bone and joint development, cell proliferation, and differentiation. The superfamily encompasses extracellular ligands, including bone morphogenetic proteins (BMPs) and growth and differentiation factors (GDFs) [35]. The BMP type I receptors (BMPR1A and BMPR1B) and one type II receptor (BMPR2) are the receptors of BMPs and are needed to form a functional complex to initiate further signaling events [36], [37]. Specific mutations in GDF5 that result in a selective inactivation of the binding to BMPR1B are a cause of BDA2 [19], [20], similarly to dominant negative mutations in the GDF5 high-affinity receptor BMPR1B itself [17], [18]. BDA2 has also been shown to be caused by mutations in GDF5 that interfere with cleavage of the GDF5 prodomain from the mature peptide [21]. BMP2 activates the BMP pathway by binding to the receptor complex [38] and plays an essential role in cartilage differentiation and bone formation, similarly to GDF5 and BMPR1B [17], [19], [39], [40], [41]. It is known that the global loss of *BMP2* in an animal results in an embryonic lethality and that BMP2 plays a center role in skeletal development, such as chondrogenesis, cartilage and bone development, the formation of bone mineral density, and limb patterning [39], [42], [43]. Therefore, our linkage results and the reported functional studies strongly support the conclusion that *BMP2* is the causal gene of the BDA2 phenotype presented in the family analyzed in the present study.

Through further investigation, we identified a 4,671 bp (Chr20: 6,809,218–6,813,888) genomic duplication downstream of *BMP2*. This duplication is located within the linked region and was cosegregated with the BDA2 phenotype in this family but not the unaffected family members nor the unrelated control individuals. Recently, Dathe *et al.* reported two microduplications (∼5.5 and ∼5.9 kb) (Chr20: 6,808,477–6,814,024 and Chr20: 6,808,129–6,814,024) in a noncoding sequence ∼110 kb downstream of *BMP2* in two European BDA2 families. Their study found that the duplication contains highly conserved sequences that constitute a cis-regulatory element regulating *BMP2* expression in the limb and that this regulating element duplication can be considered a mutational mechanism for BDA2 [22]. More recently, Su *et al.* identified a duplication of ∼4.6 kb (Chr20: 6,809,382–6,814,044) in the same region in a Chinese family affected with BDA2 [23]. Our work shows a 4,671 bp duplicated sequence in the affected individuals and a 4,507 bp overlapping region (Chr20: 6,809,382–6,813,888) with the previous studies. A microhomology *GATCA* is located at the 5′ and 3′ flanking region of this duplicated sequence. The generation of the nonrecurring rearrangement agrees well with the replication-based model called fork stalling and template switching proposed by Lee *et al.* [44]. According to this hypothesis, the microhomology identified in the present study may confuse the DNA replication machinery, causing the replication fork to stall or pause, and lead to a switch of the lagging strand to another active replication fork in physical proximity before resuming replication of the original DNA template, thereby resulting in duplication formation. Furthermore, a longer microhomology (52 bp) was identified upstream of *GATCA*, and microhomologies *GTGACC* (7 bp) [22] and *TTGCAGTGAGC* (11 bp) [23], which are associated with the duplications identified in two other BDA2 families, were also observed downstream of *GATCA* in our study. Therefore, these findings support the conclusion that this region of the genome is prone to structural changes associated with malformation of the digits and that this tendency is probably caused by the abundance of microhomologous sequences in the region.

In a functional study, Dathe *et al*. observed that a 5.5 kb tandem duplication in *BMP2* may increase the expression of *BMP2* in the limb of transgenic mouse. They speculated that the overexpression of *BMP2* may result in a deregulation of the fine-tuned BMP pathway by increasing the BMPR1A signal and in a relative decrease of BMPR1B signaling, which is compatible with the previously proposed molecular pathology of BDA2 [22]. Nevertheless, Su *et al.* revealed reduced activity of conserved 2.1 kb sequences in the duplication region in osteosarcoma U-2OS and HeLa cells through luciferase activity assays [23]. The discrepant expression caused by the duplication downstream of the *BMP2* gene *in vivo* and *in vitro* may be due to the complex regulation of *BMP2* and the other malformations observed in addition to BDA2 in some of the family members. It has been found that *BMP2* contains two promoters and that the transcription-initiation site is not common between the human and mouse *BMP2* genes, although the sequence of the *BMP2* gene is well conserved in the promoter region between the two species [45]. In the 2T3 osteoblast cell line, a proximal promoter fragment can be autoregulated by BMP2 [46]. The studies of a ∼2.7 kb *BMP2* promoter fragment have suggested that NF-κB and Gli2 can bind separately to it and thus drive *BMP2* expression in different cells [47], [48]. Moreover, the 3′-UTR of *BMP2* contains essential regulatory elements, and many serial regulatory elements are located within the large conserved non-coding regions of *BMP2* [49], [50]. Therefore, the expression of *BMP2* regulated by the duplication sequences may depend on different experimental conditions and/or interaction with other cis-regulatory elements.

BDA2 has genetic heterogeneity. The previous and current studies on BDA2 families show that three missense mutations in *BMPR1B*, two missense mutations in *GDF5*, and three duplication elements approximately 110 kb downstream of *BMP2* can separately cause the phenotype of BDA2 [17], [18], [19], [20], [21], [22], [23]. Despite the complex molecular mechanisms, these findings suggest that these three genes are all involved in the BMP signaling pathway and that the cause of BDA2 is a shift in the signaling intensity from BMPR1B to BMPR1A [25]. BMP signaling has functions in the growth plate at various stages of differentiation for cartilage and bone formation because it can interact with the BDA1-causing gene *IHH*, some TGF-β proteins, and other extracellular and nuclear factors in endochondral ossification and thereby form a molecular regulatory network [42], [51], [52], [53], [54]. Under different conditions, BMP2 can mediate BMP signaling through the canonical SMAD pathway to induce SMAD target genes, through a mitogen-activated protein kinase (MAPK) pathway to result in the induction of alkaline phosphatase (ALP) activity, or through a protein kinase C (PKC) signaling pathway to increase Bax/Bcl-2 caspase activity [37], [38], [55]. Therefore, there is no simple interpretation for BMP2 function in the phenotypes of BDA2, and further efforts to clarify the regulation and the role of BMP2 in the BMP signaling pathway and molecular network are warranted.

In summary, we identified a 4,671 bp duplication with a novel breakpoint flanked by the microhomology *GATCA* downstream of *BMP2* in a Chinese family affected with BDA2. Our findings support the conclusion that the genomic location that corresponds to the duplication region is prone to structural changes associated with malformation of the digits and that this tendency is probably caused by the abundance of microhomologous sequences in the region. Additionally, our study further exemplifies the peculiar nature of the balance of expression of the *BMP2* gene during development, and the genomic location corresponding to the duplication region is critical for the function of this gene. Moreover, our results indicate that such duplications may be considered a regulatory mutational mechanism. Therefore, similar duplications may conceivably be implemented into bioinformatic search tools to detect variants as an additional method to associate a genetic mutation with a given phenotype.

## Materials and Methods

### Subjects

The study was approved by the genetic research ethics committees of Shanghai Jiaotong University. A Chinese family with BDA2 was recruited for the study (see Figure 2). This family was from Anhui province in eastern China and does not have any distant relatives. The informed consent was written and obtained from all participants.

### Genetic analyses of *BMPR1B* and *GDF5*

Samples of peripheral blood DNA were collected from all available family members, and the DNA was isolated using standard procedures. We amplified the coding region, the splice junctions, and the promoter sequence of the *BMPR1B* and *GDF5* genes using standard protocol polymerase chain reaction (PCR). The PCR products were analyzed using 1.5% agarose gel electrophoresis. The purified PCR products were bidirectionally sequenced using PCR primers as sequencing primers and the Applied Biosystems Prism BigDye terminator cycle sequencing reaction kit. The products were evaluated using an Applied Biosystems 3100 DNA sequencer. In addition, we genotyped the flanking polymorphic microsatellite markers of *BMPR1B* and *GDF5* and performed a two-point linkage analysis using the LINKAGE 5.2 software program assuming a genetic model featuring autosomal dominance, a disease-allele frequency of 0.0001, an evenly shared allele frequency, a zero phenocopy rate, no sex difference, and full penetrance [56].

### Genome-wide scan and linkage analyses

We performed a genome-wide scan of the family using a set of 403 polymorphic microsatellite markers (ABI Prism Linkage Mapping Set version 2.5) with an average marker density of 10 centimorgans according to the Marshfield genetic linkage map. Additional markers were used for the fine mapping. The products of the PCR assays with fluorescently labeled primers were analyzed by automated capillary genotyping on MegaBACE 1000 (Amersham Pharmacia Biotech). A two-point linkage analysis was performed using the LINKAGE 5.2 software program [18], [56]. The haplotypes were reconstructed using the CYRILLIC version 2.1 software.

### Mutation analyses of *BMP2*

Following the procedures described above, we amplified and sequenced the *BMP2* gene (i.e., all exons, introns, promoter region, 5′UTR, and 3′UTR). The evolutionary conserved regions that were sequenced were Chr20: 6,811,205–6,813,071. The following evolutionary conserved sequences were identified using the publicly available web-browser program ECR [57]. The PCR products were analyzed and sequenced by standard protocols as described above.

### Duplication analyses of *BMP2*

A CGH array of human chromosome 20 was performed using a custom Roche array (NimbleGen Human CGH 385K Chromosome 20 Tiling Array). The labeling and hybridization were performed following the protocols provided by the manufacturers (NimbleGen Hybridization System 4). The array was analyzed using the NimbleGen MS 200 Microarray Scanner and NimbleScan software. A graphical overview was obtained using the SignalMap software (v1.9.0.05, NimbleGen Systems Inc). Quantitative real-time PCR (Q-PCR) with five primer pairs (P1–P5) was performed using a ViiATM 7 Real-Time PCR system and the ViiATM 7 software 1.0 (Applied Biosystems) to confirm the duplication region. PCR with the primers F7 and R7 was performed to amplify the breakpoint region, and the products were sequenced as described above. The abovementioned primers were designed by Su *et al*. [23]. The sequences were compared with the data from the UCSC Genome Browser (http://genome.ucsc.edu).

## Supporting Information

Table S1 **The summary of causing genes and phenotypes of BDA2.**
(DOC)

File S1 **Sequencing of the family number IV:3.**
(AB1)

File S2 **Sequencing of the family number IV:4.**
(AB1)

File S3 **Sequencing of the family number IV:7.**
(AB1)

File S4 **Sequencing of the family number IV:12.**
(AB1)

File S5 **Sequencing of the family number IV:15.**
(AB1)

File S6 **Sequencing of the family number V:9.**
(AB1)

File S7 **Sequencing of the family number V:10.**
(AB1)

File S8 **Sequencing of the family number V:I1.**
(AB1)

## References

[1] Temtamy SA, Aglan MS (2008) Brachydactyly. Orphanet J Rare Dis 3: 15. 18554391 10.1186/1750-1172-3-15 PMC2441618

[2] Bell J (1951) On brachydactyly and symphalangism. In Treasury of Human Inheritance Volume 5. London: Cambridge University Press: pp.1–31.

[3] Temtamy S, McKusick V (1978) The Genetics of Hand Malformations New York: Alan R Liss, INC. 215242

[4] Kornak U, Mundlos S (2003) Genetic disorders of the skeleton: a developmental approach. Am J Hum Genet 73: 447–474. 12900795 10.1086/377110 PMC1180673

[5] Gao B, Guo J, She C, Shu A, Yang M, et al (2001) Mutations in IHH, encoding Indian hedgehog, cause brachydactyly type A-1. Nat Genet 28: 386–388. 11455389 10.1038/ng577

[6] Gao B, Hu J, Stricker S, Cheung M, Ma G, et al (2009) A mutation in Ihh that causes digit abnormalities alters its signalling capacity and range. Nature 458: 1196–1200. 19252479 10.1038/nature07862

[7] Guo S, Zhou J, Gao B, Hu J, Wang H, et al (2010) Missense mutations in IHH impair Indian Hedgehog signaling in C3H10T1/2 cells: Implications for brachydactyly type A1, and new targets for Hedgehog signaling. Cell Mol Biol Lett 15: 153–176. 20024692 10.2478/s11658-009-0040-2 PMC6275863

[8] Ma G, Yu J, Xiao Y, Chan D, Gao B, et al (2011) Indian hedgehog mutations causing brachydactyly type A1 impair Hedgehog signal transduction at multiple levels. Cell Res 21: 1343–1357. 21537345 10.1038/cr.2011.76 PMC3193471

[9] Armour CM, McCready ME, Baig A, Hunter AG, Bulman DE (2002) A novel locus for brachydactyly type A1 on chromosome 5p13.3–p13.2. J Med Genet 39: 186–188. 11897820 10.1136/jmg.39.3.186 PMC1735058

[10] Oldridge M, Fortuna AM, Maringa M, Propping P, Mansour S, et al (2000) Dominant mutations in ROR2, encoding an orphan receptor tyrosine kinase, cause brachydactyly type B. Nat Genet. 24: 275–278. 10.1038/73495 10700182

[11] Schwabe GC, Tinschert S, Buschow C, Meinecke P, Wolff G, et al (2000) Distinct mutations in the receptor tyrosine kinase gene ROR2 cause brachydactyly type B. Am J Hum Genet. 67: 822–831. 10.1086/303084 PMC1287887 10986040

[12] Akbarzadeh S, Wheldon LM, Sweet SM, Talma S, Mardakheh FK, et al (2008) The deleted in brachydactyly B domain of ROR2 is required for receptor activation by recruitment of Src. PLoS One 3: e1873. 18365018 10.1371/journal.pone.0001873 PMC2268744

[13] Lehmann K, Seemann P, Silan F, Goecke TO, Irgang S, et al (2007) A new subtype of brachydactyly type B caused by point mutations in the bone morphogenetic protein antagonist NOGGIN. Am J Hum Genet 81: 388–396. 17668388 10.1086/519697 PMC1950796

[14] Polinkovsky A, Robin NH, Thomas JT, Irons M, Lynn A, et al (1997) Mutations in CDMP1 cause autosomal dominant brachydactyly type C. Nat Genet. 17: 18–19. 10.1038/ng0997-18 9288091

[15] Johnson D, Kan SH, Oldridge M, Trembath RC, Roche P, et al (2003) Missense mutations in the homeodomain of HOXD13 are associated with brachydactyly types D and E. Am J Hum Genet. 72: 984–997. 10.1086/374721 PMC1180360 12649808

[16] Mohr O, Wriedt C (1919) A New Type of Hereditary Brachyphalangy in Man Washington: Carnegie Inst: pp.5–64.

[17] Lehmann K, Seemann P, Stricker S, Sammar M, Meyer B, et al (2003) Mutations in bone morphogenetic protein receptor 1B cause brachydactyly type A2. Proc Natl Acad Sci U S A 100: 12277–12282. 14523231 10.1073/pnas.2133476100 PMC218749

[18] Lehmann K, Seemann P, Boergermann J, Morin G, Reif S, et al (2006) A novel R486Q mutation in BMPR1B resulting in either a brachydactyly type C/symphalangism-like phenotype or brachydactyly type A2. Eur J Hum Genet 14: 1248–1254. 16957682 10.1038/sj.ejhg.5201708

[19] Seemann P, Schwappacher R, Kjaer KW, Krakow D, Lehmann K, et al (2005) Activating and deactivating mutations in the receptor interaction site of GDF5 cause symphalangism or brachydactyly type A2. J Clin Invest 115: 2373–2381. 16127465 10.1172/JCI25118 PMC1190374

[20] Kjaer KW, Eiberg H, Hansen L, van der Hagen CB, Rosendahl K, et al (2006) A mutation in the receptor binding site of GDF5 causes Mohr-Wriedt brachydactyly type A2. J Med Genet 43: 225–231. 16014698 10.1136/jmg.2005.034058 PMC2563247

[21] Ploger F, Seemann P, Schmidt-von Kegler M, Lehmann K, Seidel J, et al (2008) Brachydactyly type A2 associated with a defect in proGDF5 processing. Hum Mol Genet 17: 1222–1233. 18203755 10.1093/hmg/ddn012

[22] Dathe K, Kjaer KW, Brehm A, Meinecke P, Nurnberg P, et al (2009) Duplications involving a conserved regulatory element downstream of BMP2 are associated with brachydactyly type A2. Am J Hum Genet 84: 483–492. 19327734 10.1016/j.ajhg.2009.03.001 PMC2667973

[23] Su P, Ding H, Huang D, Zhou Y, Huang W, et al (2011) A 4.6 kb genomic duplication on 20p12.2–12.3 is associated with brachydactyly type A2 in a Chinese family. J Med Genet 48: 312–316. 21357617 10.1136/jmg.2010.084814

[24] Fitch N (1979) Classification and identification of inherited brachydactylies. J Med Genet 16: 36–44. 469884 10.1136/jmg.16.1.36 PMC1012778

[25] Mundlos S (2009) The brachydactylies: a molecular disease family. Clin Genet 76: 123–136. 19790289 10.1111/j.1399-0004.2009.01238.x

[26] Venter JC, Adams MD, Myers EW, Li PW, Mural RJ, et al (2001) The sequence of the human genome. Science 291: 1304–1351. 11181995 10.1126/science.1058040

[27] Nobrega MA, Ovcharenko I, Afzal V, Rubin EM (2003) Scanning human gene deserts for long-range enhancers. Science 302: 413. 14563999 10.1126/science.1088328

[28] Kimura-Yoshida C, Kitajima K, Oda-Ishii I, Tian E, Suzuki M, et al (2004) Characterization of the pufferfish Otx2 cis-regulators reveals evolutionarily conserved genetic mechanisms for vertebrate head specification. Development 131: 57–71. 14645121 10.1242/dev.00877

[29] Uchikawa M, Takemoto T, Kamachi Y, Kondoh H (2004) Efficient identification of regulatory sequences in the chicken genome by a powerful combination of embryo electroporation and genome comparison. Mech Dev 121: 1145–1158. 15296978 10.1016/j.mod.2004.05.009

[30] Ovcharenko I, Loots GG, Nobrega MA, Hardison RC, Miller W, et al (2005) Evolution and functional classification of vertebrate gene deserts. Genome Res 15: 137–145. 15590943 10.1101/gr.3015505 PMC540279

[31] Kleinjan DA, van Heyningen V (2005) Long-range control of gene expression: emerging mechanisms and disruption in disease. Am J Hum Genet 76: 8–32. 15549674 10.1086/426833 PMC1196435

[32] Chandler RL, Chandler KJ, McFarland KA, Mortlock DP (2007) Bmp2 transcription in osteoblast progenitors is regulated by a distant 3′ enhancer located 156.3 kilobases from the promoter. Mol Cell Biol 27: 2934–2951. 17283059 10.1128/MCB.01609-06 PMC1899916

[33] Styrkarsdottir U, Cazier JB, Kong A, Rolfsson O, Larsen H, et al (2003) Linkage of osteoporosis to chromosome 20p12 and association to BMP2. PLoS Biol 1: E69. 14691541 10.1371/journal.pbio.0000069 PMC270020

[34] Sammar M, Stricker S, Schwabe GC, Sieber C, Hartung A, et al (2004) Modulation of GDF5/BRI-b signalling through interaction with the tyrosine kinase receptor Ror2. Genes Cells 9: 1227–1238. 15569154 10.1111/j.1365-2443.2004.00799.x

[35] Allendorph GP, Vale WW, Choe S (2006) Structure of the ternary signaling complex of a TGF-beta superfamily member. Proc Natl Acad Sci U S A 103: 7643–7648. 16672363 10.1073/pnas.0602558103 PMC1456805

[36] Kawabata M, Chytil A, Moses HL (1995) Cloning of a novel type II serine/threonine kinase receptor through interaction with the type I transforming growth factor-beta receptor. J Biol Chem 270: 5625–5630. 7890683 10.1074/jbc.270.10.5625

[37] Derynck R, Zhang YE (2003) Smad-dependent and Smad-independent pathways in TGF-beta family signalling. Nature 425: 577–584. 14534577 10.1038/nature02006

[38] Nohe A, Hassel S, Ehrlich M, Neubauer F, Sebald W, et al (2002) The mode of bone morphogenetic protein (BMP) receptor oligomerization determines different BMP-2 signaling pathways. J Biol Chem 277: 5330–5338. 11714695 10.1074/jbc.M102750200

[39] Tsuji K, Bandyopadhyay A, Harfe BD, Cox K, Kakar S, et al (2006) BMP2 activity, although dispensable for bone formation, is required for the initiation of fracture healing. Nat Genet 38: 1424–1429. 17099713 10.1038/ng1916

[40] Storm EE, Kingsley DM (1999) GDF5 coordinates bone and joint formation during digit development. Dev Biol 209: 11–27. 10208739 10.1006/dbio.1999.9241

[41] Baur ST, Mai JJ, Dymecki SM (2000) Combinatorial signaling through BMP receptor IB and GDF5: shaping of the distal mouse limb and the genetics of distal limb diversity. Development 127: 605–619. 10631181 10.1242/dev.127.3.605

[42] Long F, Ornitz DM (2013) Development of the endochondral skeleton. Cold Spring Harb Perspect Biol 5: a008334. 23284041 10.1101/cshperspect.a008334 PMC3579395

[43] Bandyopadhyay A, Tsuji K, Cox K, Harfe BD, Rosen V, et al (2006) Genetic analysis of the roles of BMP2, BMP4, and BMP7 in limb patterning and skeletogenesis. PLoS Genet 2: e216. 17194222 10.1371/journal.pgen.0020216 PMC1713256

[44] Lee JA, Carvalho CM, Lupski JR (2007) A DNA replication mechanism for generating nonrecurrent rearrangements associated with genomic disorders. Cell 131: 1235–1247. 18160035 10.1016/j.cell.2007.11.037

[45] Sugiura T (1999) Cloning and functional characterization of the 5′-flanking region of the human bone morphogenetic protein-2 gene. Biochem J 338 (Pt 2): 433–440. 10024520 PMC1220070

[46] Ghosh-Choudhury N, Choudhury GG, Harris MA, Wozney J, Mundy GR, et al (2001) Autoregulation of mouse BMP-2 gene transcription is directed by the proximal promoter element. Biochem Biophys Res Commun 286: 101–108. 11485314 10.1006/bbrc.2001.5351

[47] Feng JQ, Xing L, Zhang JH, Zhao M, Horn D, et al (2003) NF-kappaB specifically activates BMP-2 gene expression in growth plate chondrocytes in vivo and in a chondrocyte cell line in vitro. J Biol Chem 278: 29130–29135. 12759356 10.1074/jbc.M212296200

[48] Zhao M, Qiao M, Harris SE, Chen D, Oyajobi BO, et al (2006) The zinc finger transcription factor Gli2 mediates bone morphogenetic protein 2 expression in osteoblasts in response to hedgehog signaling. Mol Cell Biol 26: 6197–6208. 16880529 10.1128/MCB.02214-05 PMC1592805

[49] Fritz DT, Liu D, Xu J, Jiang S, Rogers MB (2004) Conservation of Bmp2 post-transcriptional regulatory mechanisms. J Biol Chem 279: 48950–48958. 15358784 10.1074/jbc.M409620200

[50] Abrams KL, Xu J, Nativelle-Serpentini C, Dabirshahsahebi S, Rogers MB (2004) An evolutionary and molecular analysis of Bmp2 expression. J Biol Chem 279: 15916–15928. 14757762 10.1074/jbc.M313531200

[51] Kronenberg HM (2003) Developmental regulation of the growth plate. Nature 423: 332–336. 12748651 10.1038/nature01657

[52] Yoon BS, Lyons KM (2004) Multiple functions of BMPs in chondrogenesis. J Cell Biochem 93: 93–103. 15352166 10.1002/jcb.20211

[53] Mackie EJ, Ahmed YA, Tatarczuch L, Chen KS, Mirams M (2008) Endochondral ossification: how cartilage is converted into bone in the developing skeleton. Int J Biochem Cell Biol 40: 46–62. 17659995 10.1016/j.biocel.2007.06.009

[54] Minina E, Kreschel C, Naski MC, Ornitz DM, Vortkamp A (2002) Interaction of FGF, Ihh/Pthlh, and BMP signaling integrates chondrocyte proliferation and hypertrophic differentiation. Dev Cell 3: 439–449. 12361605 10.1016/s1534-5807(02)00261-7

[55] Hay E, Lemonnier J, Fromigue O, Marie PJ (2001) Bone morphogenetic protein-2 promotes osteoblast apoptosis through a Smad-independent, protein kinase C-dependent signaling pathway. J Biol Chem 276: 29028–29036. 11395480 10.1074/jbc.M011265200

[56] Xing QH, Wang MT, Chen XD, Feng GY, Ji HY, et al (2003) A gene locus responsible for dyschromatosis symmetrica hereditaria (DSH) maps to chromosome 6q24.2–q25.2. Am J Hum Genet 73: 377–382. 12815562 10.1086/377007 PMC1180374

[57] Ovcharenko I, Nobrega MA, Loots GG, Stubbs L (2004) ECR Browser: a tool for visualizing and accessing data from comparisons of multiple vertebrate genomes. Nucleic Acids Res 32: W280–286. 15215395 10.1093/nar/gkh355 PMC441493

