# Supplementary material for: Identification of Duplication Downstream of BMP2 in a Chinese Family with Brachydactyly Type A2 (BDA2)
Source: PLoS One. 2014 Apr 7;9(4):e94201. doi: 10.1371/journal.pone.0094201 (PMC3978006; doi:10.1371/journal.pone.0094201)
Supplement: Table S1 — The summary of causing genes and phenotypes of BDA2. (DOC) [file pone.0094201.s001.doc]

**Table S1**. The summary of causing genes and phenotypes of BDA2

| Genes | Mutation | Subjects | Phenotype | References |
| --- | --- | --- | --- | --- |
| *BMPR1B* | I200K or R468W | two unrelated German families | All patients demonstrated BDA2 hand phenotype: shortened or medially deviated second fingers. Approximately half had some foot abnormal symptoms: deviation and shortening of the first or second toes, cutaneous syndactyly between the second and third toes, and camptodactyly. No family-specific phenotypes. | Lehmann *et al.* [17] |
|  | R486Q | Patient 1 is a German woman and patient 2 is a boy. | Patient 1 showed hand phenotypic similarities with BDC and symphalangism. Patient 2 showed typical BDA2. The foot phenotype is relative mild. | Lehmann *et al.* [18] |
| *GDF5* | L441P | A Norwegian family and one person from a Danish family | Hands were more commonly affected than the feet. Relative shortening of the second mesophalanx was predominant trait accompanied with some other abnormal symptoms. Three mutation carriers appeared clinically normal. | Seemann *et al.* [19];  Kjaer *et al.*[20] |
|  | R380Q | A family without other information | Hands were more commonly affected than the feet. The second mesophalanx was constantly shorter and the other abnormal phenotype was variable. Some affected showing an almost normal pattern while others had additional features indicating overlap with BDA1 and BDC. | Ploger *et al.* [21] |
| *BMP2* | Duplication (5.9 kb or 5.5 kb) | Two unrelated families. One is part of a large Brazilian kindred of German origin. The smaller family is of European origin as well | In the big family: The predominant trait was in accordance with BDA2 accompanied with some other abnormal symptoms. In one patient, hands appeared normal and the second toe was bilaterally short. The most severely affected individual has similarities to BDC. In the small family: only one mother and her child were investigated and both presented with characteristic BDA2 | Dathe *et al.*[22] |
|  | Duplication (4.6 kb) | A Chinese family | The patients were diagnosed with BDA2 by clinical and X-ray examinations. Three patients had triangular mesophalanx in the second toes only. One patient also had syndactyly between 3rd and 4th fingers. | Su *et al.* [23] |
|  | Duplication (4.6 kb) | A Chinese family from Anhui Province | The affected individuals had the typical phenotypes of BDA2 characteristic of medially deviated and shortened index fingers and second toes with abnormal interdigital joint formation. Triangular middle phalanges was observed in all of the affected individuals. Short stature was present in most affected adult individuals. | Current study |
